# Architectural changes in alveolar bone for dental decompensation before surgery in Class III patients with differing facial divergence: a CBCT study

**DOI:** 10.1038/s41598-020-71126-3

**Published:** 2020-09-01

**Authors:** Chung-Chen Jane Yao, Zwei-Chieng Chang, Hsiang-Hua Lai, Li-Fang Hsu, Hann-Min Hwang, Yi-Jane Chen

**Affiliations:** 1 School of Dentistry, National Taiwan University, No. 1, Chang-Te Street, Taipei City, 10048 Taiwan; 2 Divison of Orthodontics and Dentofacial Orthopedics, Department of Dentistry, National Taiwan University Hospital, Taipei City, Taiwan

**Keywords:** Health care, Risk factors

## Abstract

This study aimed to investigate alveolar bone change around mandibular anterior teeth during orthodontic decompensation in patients with skeletal Class III malocclusion and different vertical facial patterns. The records of 29 consecutive Class III patients selected from those pending two-jaw orthognathic surgery were divided into low (≤ 28°), average (30°–37°), and high (≥ 39°) mandibular plane angle (MPA) groups. The DICOM files of CBCT scans and STL files of digital dental models, taken before (T1) and after (T2) presurgical orthodontic treatment, were imported into Dolphin imaging software to reconstruct dentoskeletal images. T1 and T2 images were superimposed and analyzed for bone thickness and height at the level of root apex on each mid-sagittal slice of six mandibular anterior teeth. Differences between T1 and T2 were analyzed by non-parametric tests and mixed-effect model analysis. The results showed that the measurements of alveolar bone height generally decreased after treatment, regardless of MPA. The facial divergence, incisor irregularity, tooth site, treatment time, and change in proclination were identified as the significant factors affecting alveolar bone thickness and height during treatment. The presurgical orthodontic treatment to decompensate mandibular anterior teeth should be very careful in all MPA groups.

## Introduction

Retroclination of the mandibular incisors is a frequent manifestation of dental compensation in adults with mandibular prognathism. In surgical-orthodontic treatment for severe Class III malocclusion, dental decompensation is crucial to maximize surgical correction and normalize stomatognathic function. Inadequate dental decompensation may limit the amount of mandible setback and correction of chin prominence in skeletal Class III patients^1–3^.

The craniofacial pattern plays an important role in planning orthodontic treatment mechanics. The cortical bone thickness of dentoavleolar ridge has been reported to be greater in adults with facial hypodivergence than in those with hyperdivergence^4–6^. In a previous study, the smallest mandibular cortical bone thickness was noted in patients with hyperdivergent facial pattern, and the mandibular bone thickness differed more than bone height among long-face, short-face, and average-face groups^7^. In another study, Class III patients with high-MPA vertical pattern exhibited the smallest alveolar bone thickness compared to other types of malocclusions with vertical and sagittal jaw discrepancy^8^. Furthermore, the vertical facial pattern has been reported as a dentofacial trait significantly affecting cancellous bone thickness in the alveolar process of the mandibular incisors^9^. Other studies demonstrated that a change in tooth position alters the thickness of labial and lingual cortical plates at the level of the root apex^10^. Significant correlation has been reported between incisor inclination and morphological contour of the alveolar bone in patients with skeletal Class III malocclusion^11,12^. Cone-beam computed tomography (CBCT) images have revealed that the alveolar process of mandibular anterior teeth with more labial inclination exhibit thicker labial spongious bone^13^. Another study reported that the labial inclination of the mandibular incisors was positively correlated with labial and total alveolar bone thickness, and negatively correlated with lingual alveolar bone height^14^.

Many previous studies assessed only the condition of the alveolar bone of the mandibular incisors in orthodontic patients because of the limitations of 2D image overlapping in cephalometric analysis. Three-dimensional CBCT images allow detailed measurements of alveolar bone dimensions, including alveolar bone height and thickness, in each mandibular anterior tooth. A CBCT study conducted by Lee et al. showed that the mandibular plane angle was negatively correlated with the buccolingual alveolar bone thickness of the mandibular incisors^15^. Another CBCT study reported alveolar bone loss around the incisors in surgically treated skeletal Class III patients; the vertical alveolar bone level was more reduced in the mandibular incisors compared with maxillary incisors, particularly on the lingual side^16^. Sun et al.^14^ investigated the alveolar bone condition around the mandibular incisors in patients with skeletal Class III malocclusion. That study reported that the alveolar bone height, including the labial and lingual plates, decreased significantly after presurgical orthodontic treatment. The incisor root apex was closer to the alveolar lingual plate, whereas the alveolar bone thickness at the apex level was not significantly changed.

In addition to displaying craniofacial structures, CBCT scans enable the visualization of individual teeth and the surrounding alveolar bone in a multiplane, true-to-scale image without distortion or object-dependent non-uniform magnification^11,12,17–19^. However, metallic artifacts may be present in the CBCT images of orthodontic patients because of the radiographic scattering effect caused by metal brackets. Previous studies have shown that integrating a maxillofacial CBCT image with its corresponding laser-scanned digital dental models is suitable for generating a precise 3D dentoskeletal models with clinically acceptable registration error^20,21^. Several CBCT studies revealed that orthodontic decompensation alters the alveolar bone dimensions of the mandibular anterior teeth but without differentiating the influence of vertical facial divergence^14,22,23^.

Although the alveolar bone dimensions of mandibular incisors has been investigated in patients with skeletal Class III malocclusion^24–27^, no study has included alveolar bone change during presurgical orthodontic treatment in Class III patients with different vertical facial divergences. A previous study compared change in alveolar bone morphology at different treatment stages, whereas traditional cephalometric analysis limits the individual assessment of the mandibular anterior teeth^5^. Few studies have reported the influence of presurgical orthodontic treatment on the alveolar bone thickness and height for each of the mandibular incisors, and mandibular canines have not usually been included. The purpose of this study was to analyze the alveolar bone dimension of the six mandibular anterior teeth in skeletal Class III adult patients with different vertical facial patterns, and to investigate potential factors affecting changes in the alveolar bone dimensions in presurgical orthodontic treatment.

## Materials and methods

### Patients

This retrospective study included 29 consecutive Class III adult patients (16 men and 13 women; mean age: 21.2 ± 3.7 years) who were diagnosed with skeletal Class III malocclusion (ANB < 0°). All of the patients were treated with presurgical decompensation orthodontic treatment and two-jaw orthognathic surgery at National Taiwan University Hospital from January 2017 to December 2018. Complete high-quality records, including CBCT scans and dental models, were obtained before (T1) and at the completion of pre-surgical orthodontic treatment (T2). The study protocol was approved by the institutional review board (201705106RINB). Written informed consent was obtained from all patients. Patients with other congenital anomalies, a history of maxillofacial trauma, periodontal attachment loss, or previous root resorption were excluded from the study. The patients were categorized into three groups of low (< 28°), average (28°–39°), and high (> 39°) mandibular plane angles (MPA), indicating the difference in vertical facial divergence^28^. Table 1 shows the demographic characteristics of the patients in the three groups.

**Table 1:** Demographic characteristics of the three groups with differing facial divergence.

Variables	Low-angle (N = 8)	Normal-angle (N = 11)	High-angle (N = 10)	*p* value (Kruskal–Wallis test)
MPA (°)	26.41 ± 1.88	33.99 ± 2.46	43.28 ± 5.10	< 0.001
Age (year)	21.25 ± 3.50	20.00 ± 2.41	23.20 ± 7.33	NS
Sex (M/F)	5/3	6/5	5/5	NS
ANB (°)	− 10.76 ± 2.48	− 12.49 ± 5.79	− 11.86 ± 4.84	NS
Wits value (mm)	− 3.41 ± 1.88	− 3.46 ± 2.32	− 3.03 ± 3.49	NS
L1-MP (°)	81.54 ± 5.08	79.39 ± 5.95	76.03 ± 6.45	NS
Space	2 (25%)	3 (27%)	3 (30%)	NS
Presence of M3	3 (37%)	3 (27%)	4 (40%)	NS
IRI (1/2/3)	3/2/3	6/2/3	3/4/3	NS
Treatment time (mths)	10.50 ± 3.70	11.91 ± 4.18	10.30 ± 4.11	NS

Data are presented as mean and standard deviation.

NS, non-significant; Space, space available due to tooth missing, interdental spacing, or extraction of tooth other than third molar; M3, mandibular third molar; IRI, incisor irregularity index (1: mild, 2: moderate, 3: severe).

Although these patients were treated in a postgraduate orthodontic clinic, all patients received 0.018 × 0.025″ slot edgewise appliances. The wire sequence in the treatment followed the common principles in biomechanics. At the initial stage, 0.016″ Ni–Ti wires were routinely used, followed by a sequential wire change from 0.016″ stainless wire, 0.016 × 0.022″ copper Ni–Ti wires, to 0.016 × 0.022″ stainless steel wires for complete alignment and leveling. The presurgical treatment time was 11 months on average.

**Figure 1:**
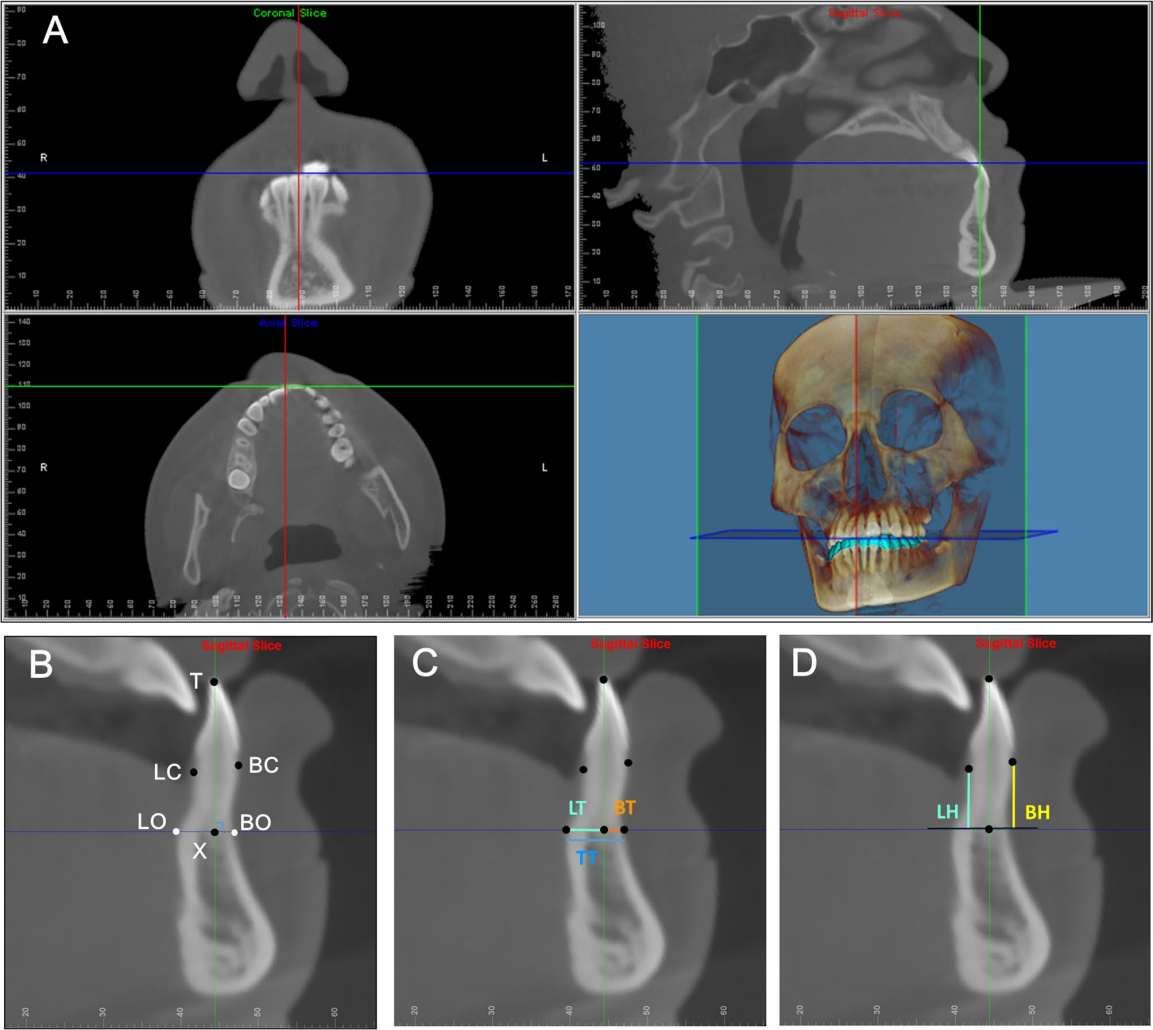
Landmarks and measurements on the midsagittal slices of the mandibular anterior teeth. (**A**) Integration of CBCT images and digital dental models improved the display of dentoalveolar structures. (**B**) Landmarks on the incisal edge midpoint/cusp tip (T), root apex (X), buccal alveolar crest (BC), lingual alveolar crest (LC), outermost point of the buccal cortex (BO), and outermost point of the lingual cortex (LO). (**C**) Alveolar bone measurements of buccal thickness (BT), lingual thickness (LT), and total thickness. (**D**) Alveolar bone measurements of buccal height (shortest distance from BC to the axis of the abscissa, BH) and lingual height (shortest distance from LC to the axis of the abscissa, LH).

Complete high-quality records, including CBCT scans and dental models, were obtained before (T1) and at the completion of presurgical orthodontic treatment (T2). The dental models at T1 and T2 were scanned using 3Shape TRIOS (Cart, 3Shape A/S, Copenhagen, Denmark), and the data were exported as STL files. The CBCT images were obtained using a Morita 3D Accuitomo XYZ Slice View Tomograph (Model: MCT-1, Type: EX 1/2 F17) (J. Morita MFG. Corp., Kyoto, Japan). The machine setting parameters were 90 kVp, 5 mA, EFOV scan mode, 35 s, and an isotropic 0.25-mm voxel size. The scan data were exported in DICOM format and further processed using Dolphin Imaging Premium 3D software (Ver. 11.95; Dolphin Imaging and Management Solutions, Chatsworth, CA, USA). The 3D CBCT images were re-oriented using three reference planes based on the nasion, sella turcica, basion, porion, and orbitale landmarks, as described in our previous study^29^. Afterwards, the scanned data of dental models were combined with the CBCT data to reconstruct dentoskeletal models (Fig. 1A). To improve the integration procedures, the brackets on T2 models were removed from the regions of interest. Furthermore, the T1 and T2 dentoskeletal models were superimposed using Dolphin 3D voxel-based superimposition on the mandibular symphysis^30^.

### Measurement of alveolar bone thickness and height

The alveolar bone thickness and height were determined for 174 mandibular anterior teeth in 29 patients. The images acquired for analysis were the midsagittal slice of each individual tooth that passed the root apex and the midpoint of the crown in the labial aspect (Fig. 1A). The midpoint of the incisal edge/cusp tip (T) and root apex (X) were identified by meticulously checking the different multiplanar views. Moreover, four landmarks were identified on the outermost of the buccal cortex (BO) and the lingual cortex (LO) at the level of the apex, and the buccal and lingual alveolar crests (BC, LC). X was defined as the origin, T–X as the axis of the ordinates, and BO–LO as the axis of the abscissa (Fig. 1B). All image processing and landmark identification was performed on an LCD monitor (ViewSonic 21.5″VA2248M-LED; ViewSonic Corp., CA, USA) by one operator (H.-M. H). The corresponding 3D coordinates (x, y, z) of all the landmarks were then sequentially input to Excel (Microsoft Excel 2010; Microsoft, Redmond, WA, USA) for calculation of five alveolar bone dimensions, including buccal thickness (BT, the perpendicular distance from BO to the tooth axis), lingual thickness (LT, the perpendicular distance from LO to the tooth axis), total thickness (TT, the distance between BO and LO), buccal height (BH, the perpendicular distance from BC to the axis of the abscissa), and lingual height (LH, the distance from LC to the axis of the abscissa; Fig. 1C, D). Furthermore, the difference in measurements at T1 and T2 were calculated to identify the alveolar bone dimensional changes incurred during presurgical orthodontic treatment. These items included the difference in buccal thickness (D_BT), difference in lingual thickness (D_LT), difference in total thickness (D_TT), difference in buccal height (D_BH), and difference in lingual height (D_LH). A positive sign denoted an increase in alveolar bone measurements, and a negative sign denoted a decrease.

### Assessment of tooth movement

**Figure 2:**
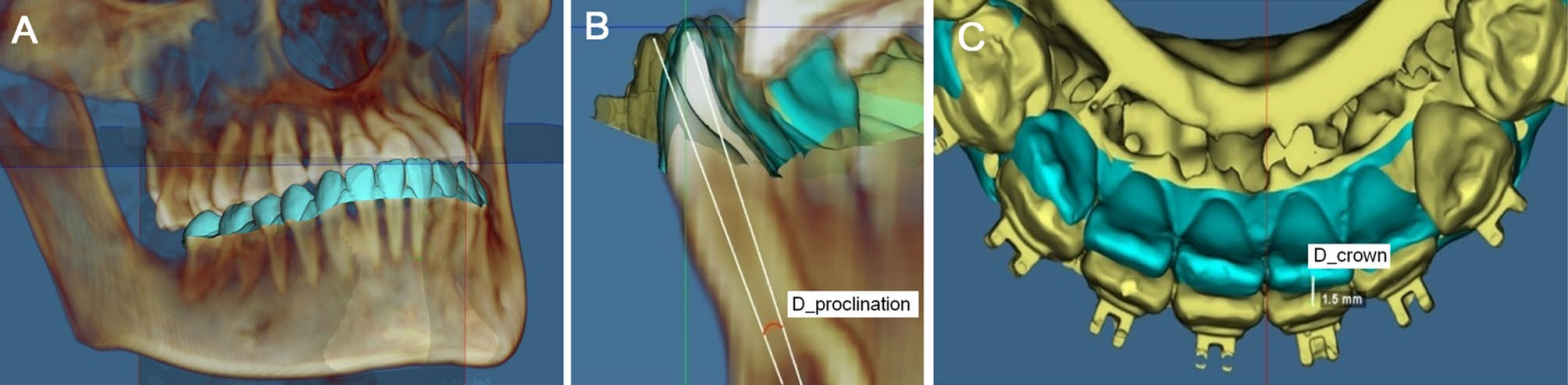
Tooth movements of the mandibular anterior teeth were analyzed on two sets of CBCT scans obtained before (T1) and after (T2) decompensation. (**A**): Three-dimensional dentoskeletal model at T1. (**B**) Difference in the tooth axis (D_proclination) superimposed on the mandibular bony structure. (**C**) Distance of crown movement (D_crown) analyzed by referring to the mandibular occlusal plane at T1. (blue color: T1, yellow color: T2).

To quantitatively assess orthodontic tooth movement incurred during treatment, the mesiobuccal cusp tips of the bilateral mandibular first molars and the midpoint between the incisal edges of the mandibular central incisors were used to determine the mandibular occlusal plane (MOP). The plane passing the midpoints of the left and right central incisor edges and perpendicular to the MOP was defined as the vertical reference plane. To analyze the orthodontic movement of mandibular anterior teeth, the T1 and T2 dentoskeletal models were superimposed with registration on the mandibular symphysis^31^. The “transfer orientation” algorithm in Dolphin software was used to analyze difference in tooth position at T1 and T2 by using the MOP at T1 as the reference plane^22^. Difference in the proclination of tooth axis (D_proclination) and difference in the incisal-edge position (D_crown) were analyzed separately for each individual tooth (Fig. 2). The use of the MOP at T1 helped eliminate the influence of occlusion change and alteration of the mandiblular position, which typically occurs during presurgcial orthodontic treatment. Moreover, difference in the root length at T1 and T2 was divided by the root length at T1 and denoted as DTLp (percentage of difference in root length) for evaluation of root resorption incurred during the treatment.

Furthermore, several patient-related clinical variables were recorded, including space available as a result of missing tooth, interdental space, and extraction of teeth other than third molars, incisor irregularity index modified from Little’s irregularity index (IRI, mild: < 4, moderate: 4–8, severe > 8)^32^, and treatment time. In addition to the variables mentioned previously, three commonly used cephalometric measurements, ANB, Wit value, L1-MP (labial inclination of central incisor) were recorded.

### Statistical analysis

To evaluate measurement error, 10 pairs of CBCT images at T1 and T2 were randomly selected for integration with their digital dental models, and repeatedly analyzed at an interval of 2 weeks. Random errors were calculated according to Dahlberg’s formula. Statistical analysis was performed using SAS 9.4 (SAS Institute Inc., Cary, NC, USA). The measurements of the left and right mandibular anterior teeth were averaged and expressed as the mean and standard deviation according to the tooth site (central incisor, lateral incisor, and canine).

The Kruskal–Wallis test and post hoc Dunn’s test were performed to check for differences of the measurements before treatment (T1) among the low-, normal-, and high-MPA groups. The Wilcoxon signed-rank test was then used to check for differences between the T1 and T2 measurements. Linear mixed-effect model analysis was performed to identify factors affecting the alveolar bone measurements. The potential factors included sex, age, MPA group, ANB, Wit value, L1-MP, treatment time, space available, IRI (incisor irregularity index), presence of M3 (third molars), TT (total alveolar bone thickness at T1), tooth site (1: central incisor, 2: lateral incisor, 3: canine), and change of tooth axis (D_proclination). Furthermore, all of the significant factors identified in the univariate analysis were entered into the multivariate analysis. A *p* value of < 0.05 was considered statistically significant.

### Ethical approval

This study protocol was approved by the Institutional Review Board of National Taiwan University Hospital (201705106RINB). All procedures performed in studies involving human participants were in accordance with the ethical standards of the institutional research committee and with the 1964 Helsinki declaration and its comparable ethical standards.

## Results

Table 1 shows the distribution and demographic characteristics of the patients according to their vertical facial patterns. No statistically significant difference was observed in terms of age, sex, Wit’s value, ANB, L1-MP, space, presence of third molars, incisor irregularity index (IRI), and treatment time among the low-, normal-, and high-MPA groups. The mean measurement error of alveolar bone thickness ranged from 0.15 ± 0.05 mm to 0.21 ± 0.04 mm, and that of alveolar bone height was 0.47 ± 0.04 mm to 0.49 ± 0.05 mm. The random errors in the cephalometric measurements were within 0.5° (range: 0.43°–0.49°). The mean percentage of the difference in root length at T1 and T2 (DTLp) was 2.13% in the central incisors, 2.53% in the lateral incisors, and 1.36% in the canines.

### Alveolar bone thickness and height among patients with differing facial divergence

Table 2 shows the alveolar bone measurements of the three groups before treatment (T1). The high-MPA patients had lower alveolar bone thickness and height than the normal- and low-MPA patients did in all mandibular incisors and canines. The lowest values were generally noted in the high-MPA group, followed by the normal-angle group; the highest values were measured in the low-MPA patients (Supplementary Fig. S1). The Kruskal–Wallis test revealed that the lingual thickness (LT), total thickness (TT), and lingual heights (LH) of alveolar bone at all tooth sites were significantly greater in the low-MPA group than in the high-MPA group. However, the measurements of buccal thickness (BT) and buccal height (BH) did not show any significant group difference at any tooth site.

**Table 2:** Median alveolar bone thickness and height of the mandibular anterior teeth before treatment.

Measurement	Median (IQR)			*p* value (Kruskal–Wallis test)
	Low-angle (N = 8)	Normal-angle (N = 11)	High-angle (N = 10)	
**Central incisor**				
BT	2.17 (0.51)	1.89 (0.67)	2.10 (0.77)	NS
LT	**5.30** (2.00)^a^	3.60 (1.26)	**3.24** (2.01)	**0.004**
TT	**7.36** (1.82)^a^	5.45 (1.83)	**5.34** (2.73)	**0.013**
BH	9.00 (1.76)	8.25 (2.35)	8.03 (5.50)	NS
LH	**7.93** (3.23)^a^	5.50 (3.45)	**4.23** (2.65)	**0.028**
**Lateral incisor**				
BT	2.78 (1.40)	1.97 (1.01)	1.79 (0.94)	NS
LT	**5.95** (1.67)^a^	4.65 (2.72)	**3.18** (1.85)	**0.002**
TT	**7.94** (2.05)^a^	6.28 (2.07)	**5.22** (2.45)	**0.001**
BH	9.93 (2.50)	9.45 (1.70)	8.90 (3.50)	NS
LH	**8.95** (1.78)^a^	8.50 (3.40)	**6.23** (3.80)	**0.004**
**Canine**				
BT	3.40 (1.76)	3.61 (2.30)	3.34 (2.22)	NS
LT	**6.54** (2.77)^a^	5.32 (2.84)	**4.29** (2.52)	**0.008**
TT	**10.10** (0.73)^a^	9.29 (2.90)	**8.04** (5.14)	**0.005**
BH	9.83 (3.99)	8.60 (8.35)	5.25 (4.25)	NS
LH	**11.00** (2.49)^a^	11.00 (2.10)	**8.85** (2.60)	**0.024**

BT, buccal thickness; LT, lingual thickness; TT, total thickness; BH, buccal height; LH, lingual height; IQR, inter-quartile range; NS, non-significant.

^a^Significant difference between low-angle and high-angle groups.

Statistically significant items are indicated in bold.

### Changes in alveolar bone thickness and height during presurgical decompensation

Table 3 shows the differences in measurements between T1 and T2. The results of the Wilcoxon signed-rank test revealed significant differences in all of the measurements for overall changes at three different tooth sites. The measurements of D_BT were positive at either the incisors or canines. However, negative values were noted for all of the other measurements, indicating a general trend in the change of alveolar bone dimensions. To determine the effect of facial divergence, the measurement differences among the three MPA groups were analyzed for all tooth sites. The results of the Kruskal–Wallis test showed that the changes in the alveolar bone thickness of the central and lateral incisors were not statistically significant among the low-, normal-, and high-MPA groups. The D_BT of the canines was significantly greater in the low-MPA group than in the high-MPA group. Similar trends were also observed at the incisor area but without reaching significant levels. Similar to D_LT and D_TT, negative values of D-BH and D-LH were consistently noted for all tooth sites in the three groups with different vertical facial patterns. The variation in measurements was greater in alveolar height (D-BH, D-LH) than in alveolar thickness (D-BT, D-LT, and D-TT), as shown by the boxplots in Supplementary figure S2.

**Table 3:** Change in alveolar bone thickness and height after treatment.

Measurement	Overall (N = 29)	*p *value^a^	Median (IQR)			*p *value^b^
			Low-angle (N = 8)	Normal-angle (N = 11)	High-angle (N = 10)	
**Central incisor**						
D_BT	0.22 (1.09)	0.050	1.31 (1.33)	0.00 (1.07)	0.11 (0.52)	NS
D_LT	− 1.25 (1.23)	< 0.001	− 1.70 (1.80)	− 0.87 (1.50)	− 1.17 (0.91)	NS
D_TT	− 0.76 (0.87)	< 0.001	− 0.46 (1.30)	− 0.62 (0.90)	− 0.87 (0.56)	NS
D_BH	− 2.70 (0.45)	< 0.001	− 1.93 (4.11)	− 3.20 (5.30)	− 2.38 (2.85)	NS
D_LH	− 1.0 (2.95)	0.003	− 1.93 (3.81)	− 1.00 (4.70)	− 0.98 (2.85)	NS
D_crown	1.35 (2.05)	< 0.001	1.53 (2.00)	1.00 (3.85)	1.33 (2.48)	NS
D_proclination	7.74 (6.05)	< 0.001	9.31 (5.06)	9.38 (5.58)	8.36 (7.54)	NS
**Lateral incisor**						
D_BT	0.41 (1.03)	0.001	1.08 (1.11)	0.44 (1.09)	0.33 (0.55)	NS
D_LT	− 1.07 (1.13)	< 0.001	− 1.33 (1.75)	− 0.55 (1.69)	− 0.58 (1.04)	NS
D-TT	− 0.28 (0.94)	< 0.001	− 0.10 (1.57)	− 0.54 (0.96)	− 0.23 (0.34)	NS
D_BH	− 3.45 (3.50)	< 0.001	− 1.08 (2.95)	− 3.70 (1.70)	− 3.85 (3.25)	NS
D_LH	− 1.35 (4.08)	0.001	− 0.85 (3.44)	− 2.43 (3.35)	0.40 (5.45)	NS
D_crown	0.95 (1.96)	< 0.001	1.20 (1.18)	1.55 (3.60)	0.65 (2.20)	NS
D_proclination	6.04 (6.03)	< 0.001	6.43 (3.76)	5.90 (9.98)	5.02 (7.47)	NS
**Canine**						
D_BT	0.66 (0.88)	0.006	**0.97** (0.95)^#^	0.29 (0.62)	**0.08** (0.47)	**0.009**
D_LT	− 0.85 (0.91)	< 0.001	− 1.55 (1.22)	− 1.04 (1.15)	− 0.76 (0.67)	NS
D_TT	− 0.21 (0.93)	0.010	− 0.08 (1.56)	− 0.32 (1.12)	− 0.28 (0.64)	NS
D_BH	− 0.60 (3.30)	0.002	− 0.20 (2.61)	− 0.60 (7.85)	− 1.25 (3.50)	NS
D_LH	− 1.28 (2.51)	< 0.001	− 0.83 (1.44)	− 1.45 (5.20)	− 1.65 (2.45)	NS
D_crown	0.75 (2.35)	0.030	0.80(1.61)	1.55 (2.70)	0.18 (1.68)	NS
D_proclination	5.97 (6.43)	< 0.001	5.37 (5.65)	7.05 (10.05)	5.14 (6.34)	NS

D_BT, difference in buccal thickness; D_LT, difference in lingual thickness; D_TT, difference in total thickness; D_BH, difference in buccal height; D_LH, difference in lingual height, IQR, inter-quartile range; NS, non-significant in post-hoc inter-group comparison.

^a^Wilcoxon signed-rank test.

^b^Kruskal–Wallis test.

#Significant difference between low-angle and high-angle groups.

Statistically significant items are indicated in bold.

### Potential risk factors identified in univariate and multivariate linear mixed-effect models

Table 4 shows the results of univariate mixed-effect linear models for the respective measurements. The beta coefficient indicated the effect of an individual group relative to the reference group with a coefficient of 1. The significant factors affecting the buccal alveolar bone thickness change (D_BT) included MPA group and tooth axis proclination (D_proclination). Compared to the low-MPA group, the D_BT was − 0.746 mm (*p* = 0.039) and − 1.162 mm (*p* = 0.003) in the average- and high-MPA groups, respectively, indicating a decrease in BT in the average- and high-MPA groups compared with the low-MPA group. Moreover, D_BT increased by 0.099 mm for a 1° increase in D_proclination (*p* < 0.0001). In the multivariate mixed-effect linear model (Table 5), D_BT was − 0.734 mm (*p* = 0.008) and − 0.947 mm (*p* = 0.001) for the average- and high-MPA groups, respectively, compared with the low-MPA group, and increased by 0.097 mm for a 1° increase in D_proclination (*p* < 0.0001).

**Table 4:** Univariate linear mixed-effect models for changes in alveolar bone thickness and height (crude models).

Variable	D_BT			D_LT			D_TT		
	Beta coefficient	SE	*p* value	Beta coefficient	SE	*p* value	Beta coefficient	SE	*p* value
Sex	0.515	0.305	0.103	− 0.225	0.372	0.550	0.284	0.209	0.186
Age	− 0.024	0.032	0.464	0.034	0.038	0.382	0.009	0.022	0.686
**MPA group**									
Average vs. low	− 0.746	0.343	0.039	0.659	0.431	0.138	− 0.086	0.267	0.752
High vs. low	− 1.162	0.350	0.003	0.966	0.438	0.037	− 0.142	0.272	0.605
**ANB**	− 0.047	0.034	0.182	0.074	0.039	0.065	0.028	0.023	0.228
**Wit value**	− 0.069	0.061	0.271	0.137	0.069	0.057	0.064	0.041	0.128
**L1-MP**	− 0.008	0.026	0.778	0.034	0.030	0.273	0.022	0.017	0.211
**Treatment time**	0.073	0.039	0.071	− 0.094	0.045	0.044	− 0.024	0.028	0.400
**Space**	− 0.546	0.341	0.121	0.176	0.415	0.675	− 0.384	0.230	0.106
**IRI**									
Moderate vs. mild	− 0.122	0.388	0.757	− 0.130	0.436	0.768	− 0.246	0.237	0.308
Severe vs. mild	0.114	0.377	0.766	− 0.721	0.423	0.101	− 0.606	0.232	0.015
**Presence of M3**	− 0.442	0.325	0.184	0.519	0.379	0.182	0.984	0.223	0.663
**TT**	− 0.019	0.051	0.715	− 0.038	0.057	0.505	− 0.037	0.043	0.402
**Tooth site**									
2 vs. 1	0.080	0.179	0.655	0.245	0.185	0.189	0.322	0.179	0.078
3 vs. 1	− 0.044	0.222	0.844	0.544	0.234	0.024	0.460	0.203	0.028
**D_proclination**	0.099	0.019	< 0.0001	− 0.147	0.018	< 0.0001	− 0.041	0.018	0.024

Variable	D_BH			D_LH					
	Beta coefficient	SE	*p* value	Beta coefficient	SE	*p* value			
Sex	− 0.109	0.613	0.861	0.355	0.820	0.668			
Age	0.018	0.063	0.776	0.038	0.083	0.656			
**MPA group**									
Average vs. low	− 1.748	0.691	0.018	− 1.065	0.989	0.292			
High vs. low	− 0.743	0.711	0.306	− 0.189	1.015	0.854			
**ANB**	− 0.073	0.065	0.273	0.142	0.085	0.105			
**Wit value**	− 0.122	0.118	0.311	− 0.087	0.160	0.592			
**L1-MP**	− 0.061	0.050	0.232	0.077	0.066	0.254			
**Treatment time**	− 0.012	0.080	0.880	− 0.318	0.087	0.001			
**Space**	− 0.516	0.680	0.455	− 1.799	0.877	0.049			
**IRI**									
Moderate vs. mild	− 0.621	0.730	0.403	− 0.424	0.964	0.664			
Severe vs. mild	0.100	0.713	0.890	− 1.320	0.941	0.173			
**Presence of M3**	− 0.253	0.638	0.695	0.172	0.856	0.843			
**TT**	0.279	0.134	0.039	− 0.074	0.151	0.623			
**Tooth site**									
2 vs. 1	− 0.212	0.683	0.757	− 0.723	0.537	0.184			
3 vs. 1	0.955	0.701	0.179	− 0.470	0.651	0.473			
**D_proclination**	0.054	0.053	0.313	− 0.002	0.061	0.972			

D_BT, difference in buccal thickness; D_LT, difference in lingual thickness; D_TT, difference in total thickness; D_BH, difference in buccal height; D_LH, difference in lingual height.

**Table 5:** Multivariate linear mixed-effect models for change of alveolar bone thickness and height after treatment (adjusted models).

Measurement	Variable	Beta coefficient	SE	*p *value
**D_BT**	**MPA group**			
	Average vs. low	− 0.734	0.255	0.008
	High vs. low	− 0.947	0.262	0.001
	**D_proclination**	0.097	0.017	< 0.0001
**D_LT**	MPA group			
	Average vs. low	0.677	0.333	0.053
	High vs. low	0.720	0.338	0.043
	**D_proclination**	− 0.137	0.018	< 0.0001
	**Tooth site**			
	2 vs. 1	0.177	0.139	0.209
	3 vs. 1	0.301	0.176	0.093
	**Treatment time**	− 0.019	0.037	0.603
**D_TT**	**Tooth site**			
	2 vs. 1	0.316	0.172	0.073
	3 vs. 1	0.417	0.193	0.035
	**IRI**			
	Moderate vs. mild	− 0.170	0.238	0.482
	Severe vs. mild	− 0.576	0.232	0.020
	**D_proclination**	− 0.033	0.017	0.054
**D_BH**	**MPA group**			
	Average vs. low	− 1.288	0.720	0.085
	High vs. low	0.066	0.817	0.936
	**TT**	0.279	0.146	0.061
**D_LH**	**Treatment time**	− 0.284	0.092	0.005
	**Space**	− 0.880	0.808	0.286

D_BT, difference in buccal thickness; D_LT, difference in lingual thickness; D_TT, difference in total thickness; D_BH, difference in buccal height; D_LH, difference in lingual height.

The significant factors affecting lingual alveolar bone thickness (D_LT) included MPA group, treatment time, tooth site, and D_proclination (Table 4). Compared to the low-MPA group, the D_LT increased by 0.966 mm (*p* = 0.037) in the high-MPA group, and increased by 0.544 mm (*p* = 0.024) in the canine compared with the central incisors. The increase in treatment time (1-month) induced a small but statistically significant decrease in D_LT (− 0.094 mm, *p* = 0.044). The increase in D_proclination showed a small but statistically significant decrease in D-LT (− 0.147 mm, *p* < 0.0001). However, the effects of treatment time and tooth site were non-significant in the multivariate mixed-effect models (Table 5), which showed an increase of 0.72 mm in D_LT in the high-MPA group (*p* = 0.043) relative to the low-MPA group, and a decrease of 0.137 mm for 1° increase in D_proclination (*p* < 0.0001).

The significant factors affecting total alveolar bone thickness (D_TT) included IRI, tooth site, and D-proclination (Table 4). The D_TT decreased in the patients with more incisor crowding (− 0.606 mm, severe versus moderate IRI) (*p* = 0.015). The D_TT increased (0.46 mm, *p* = 0.028) in the canines compared with the central incisors, whereas it decreased (− 0.041 mm, *p* = 0.024) with tooth proclination (D_proclination). In the multivariate mixed-effect model, the effect of D_proclination on D_TT was non-significant (Table 5), whereas D_TT showed an increase in the canines (0.417 mm, *p* = 0.035) relative to the central incisors, and decreased in the patients with severe incisor irregularity (− 0.576 mm, *p* < 0.020) compared to moderate crowding.

The significant factors affecting the changes in alveolar bone height included MPA group and TT for D_BH, and treatment time as well as presence of space for D_LH (Table 4). Compared to the low-MPA group, D_BH decreased by − 1.748 mm (*p* = 0.018) in the average-MPA group, but increased with the initial alveolar bone thickness (0.279 mm, *p* = 0.039). However, these two variables did not reach statistical significance in the multivariate mixed-effect models (Table 5). Regarding D-LH (Table 4), the increase in treatment time (1-month) induced a significant decrease in D_LH (− 0.318 mm, *p* = 0.001). The presence of space induced a decrease in D_LH (− 1.799 mm, *p* = 0.049). In the multivariate mixed-effect models (Table 5), only the treatment time was the significant variable affecting lingual height (D-LH, − 0.284 mm, *p* = 0.005).

## Discussion

In patients with skeletal Class III malocclusion and mandibular prognathism, insufficient incisor decompensation can compromise surgical outcomes. However, extensive tooth movement may be accompanied by a negative effect on the alveolar bone housing of the anterior teeth (e.g., bone dehiscence and fenestration). Special attention should be focused on the alveolar bone around the mandibular anterior teeth in treating Class III patients with mandibular prognathism. Evaluating and identifying the clinical factors associated with changes in alveolar bone dimensions in presurgical orthodontic decompensation is also crucial.

Our present study revealed that in skeletal Class III patients, the alveolar bone dimensions of the mandibular anterior teeth differed among patients with different vertical facial patterns. All of the measurements investigated, including the alveolar bone thickness at the level of root apex (BT, LT, TT) and the alveolar crest height (BH, LH), were smaller in the high-MPA patients than in the low-MPA patients. Interestingly, the group differences were statistically significant on the lingual side (LT, LH), but not on the labial side (BT, BH). After presurgical decompensation, the measurements for LT, TT, BH, and LH decreased regardless of facial divergence, and these changes reached the level of statistical significance. By using linear mixed-effect model analysis, five variables including MPA group, IRI (incisor irregularity index), tooth site, treatment time, and change in proclination (D_proclination) were identified as significantly predictive of alveolar bone measurements after treatment. The significant variables for each measurement item were MPA group and D_proclination for D_BT, MPA group and D_proclination for D_LT, tooth site and IRI for D-TT, and treatment time for D_LH. These results implied that presurgical orthodontic treatment should be meticulously and efficiently provided for the mandibular anterior teeth, particularly in patients with high MPA.

In patients with limited space deficiency of the mandibular incisors, labial tipping movement can help gain space for tooth alignment and maximize anterior crossbite for mandibular setback. Therefore, non-extraction therapy is a common strategy for mandibular dentition in the presurgical orthodontic treatment of Class III patients. However, more crowding of the mandibular anterior teeth requires more labial tipping of the incisors, possibly leading to a higher risk of alveolar bone loss. Previous studies on anterior alveolar bone dimensions included patients with mild crowding in the mandibular arch^14,15,22,23^. In the current study, the severity of space deficiency of the mandibular anterior teeth was categorized according to the irregularity index modified from Little’s irregularity index^17^. Multivariate mixed-effect models showed that the IRI was significantly predictive of the change in total alveolar bone thickness (D_TT). The total alveolar bone thickness after treatment was smaller in patients with severe IRI than those with moderate IRI (Table 5).

Significant associations have been identified among gingival biotypes, tooth inclination, and alveolar plate thickness surrounding mandibular incisors in skeletal Class III patients^33^. Conventional cephalometric study reported that vertical facial pattern did influence presurgical decompensation of the mandibular incisors in skeletal Class III malocclusion^5^. Previous CBCT studies showed presurgical orthodontic treatment-related alveolar bone loss around the incisors in skeletal Class III patients^14,16^. Forward movement of the mandibular incisors during presurgical decompensation causes the root apex of the incisors to be closer to the lingual alveolar plate and leads to loss of vertical alveolar height^14,16^. A previous study reported significant decreases in labial height (1.43 and 1.58 mm) and lingual height (0.94 mm and 0.98 mm) for central and lateral incisors, respectively^22^. The present study also revealed significant decreases in labial height (D_BH, − 2.70, − 3.45, − 0.6 mm) and lingual height (D_LH, − 1.0, − 1.35, − 1.28 mm) for the mandibular central incisors, lateral incisors, and canine. However, no significant difference in D_BH or D_LH was noted among the low-, normal- and high-MPA groups.

Our study demonstrated the significant treatment changes in total alveolar bone thickness at the level of the root apex of the six mandibular anterior teeth. These results conflict with those of a previous study that reported no significant change in total alveolar bone thickness^14^. Another finding of decreasing bone thickness on the labial and lingual aspects is consistent with other studies^16,22^. Geometrically, uncontrolled labial tipping of mandibular anterior teeth increases tooth axis proclination, leading to an increase in BT and decrease in LT. This implies that bone thickness decreased on the side toward which a root had moved and increased on the opposite side. In other words, the incisor root apex is closer to the lingual side of alveolar bone after presurgical orthodontic treatment. Therefore, labial proclination of mandibular anterior teeth would lead to an increase in BT, whereas a decrease in BT might imply alveolar bone loss incurred during orthodontic decompensation. In the current study, a significant correlation between D_proclination and D_BT (Pearson correlation coefficient = 0.753) was noted in the low-MPA group, but not in the average- and high-MPA groups. These findings are consistent with the results of multivariate analysis (Table 5, adjusted mixed-effect models), which showed the significant effect of MPA group and tooth proclination on the variation of D_BT and D_LT. An increase in decompensation of 1° (D_proclination) for labial tipping increased BT by 0.097 mm (D_BT) and reduced LT by 0.0137 mm (D_LT). The influence on the lingual thickness was statistically significant, but may not be clinically significant.

According to the multivariate analysis, the tooth site, incisor irregularity index, and change in proclination were significant in affecting the total alveolar bone thickness after decompensation (D_TT). Surprisingly, none of the investigated variables significantly influenced the buccal bone height after treatment (D_BH), and the change in LH (D_LH) was significantly affected only by the treatment time. These results implied that some factors not investigated in the present study, such as lip pressure and gingival biotype, may influence the buccal bone height during treatment. In presurgcial orthodontics for Class III malocclusion, more crowding and lingual inclination of the mandibular incisors typically requires more dental decompensation, thus leading to an increase in treatment time. After adjusting the effect of space, the decrease in lingual alveolar bone height was 0.284 mm if the treatment time was increased by 1 month (Table 5, adjusted models).

Alveolar plate thickness is affected by a variety of factors, e.g. tooth position, tooth inclination, and gingival biotype^33,34^. Previous CBCT studies revealed the significant correlation between the changes of tooth inclination and alveolar bone thickness in orthodontic patients^10,22^. Our study revealed that the alveolar height of mandibular anterior teeth was lower on the lingual side than on the labial side before treatment, and decreased on both the labial and lingual sides after decompensation. Previous studies have reported that in skeletal Class III patients, the lingual inclination of the mandibular incisors is significantly correlated with vertical facial divergence, and is smaller in high-MPA patients than in normal- or low-MPA patients^5,15,26^. The patients included in the current study exhibited severe skeletal Class III malocclusion (ANB: − 11.8° (− 19.6° to − 1.9°), and thus their mandibular incisors exhibited obvious lingual tipping (L1-MP: 78.8° (68.6°–90.1°)). All of the patients in this study were treated with non-extraction therapy for mandibular dentition. The extraction of mandibular third molars can often provide space for distalization of the posterior teeth to help relieve crowding in the middle segment of the mandibular arch. The adjusted mixed-effect models revealed that the IRI was significantly predictive of TT after treatment (D_TT), indicating a decrease in total alveolar bone thickness induced by mandibular anterior teeth with more crowding (Table 5). The multivariate analysis also showed that the presurgcial orthodontic treatment time was the only significant variable affecting treatment change in lingual alveolar bone height (D_LH). Surprisingly, none of the investigated variables were significantly predictive of the labial alveolar bone height in multivariate mixed-effect models (Table 5). In this study, all of the mandibular anterior teeth were moved labially with an increased labial proclination of the tooth axis. The effect of tooth site on the total alveolar thickness after treatment (D_TT) was also statistically significant, with an increase of 0.417 mm in the canines relative to the central incisors, which might have resulted from measurements of the bone thickness at the anatomic midpoint of the canines, a site lateral to the direction of decompensation, not the direct compression zone in the direction of movement (as was the case for the incisors).

The limits of alveolar housing and the risk of developing bone dehiscence have to be taken account when planning presurgical orthodontic treatment. CBCT is increasingly used as a tool for linear measurements of dental-alveolar structures, even there is a risk of overestimating fenestrations and dehiscence^35^. The presence of fenestration/ dehiscence was most prevalent in the mandibular incisors and canines^36,37^. Moreover, labial malposition of canines is frequently seen in the mandibular dentition with lingually inclined and crowded incisors. In addition to appropriate biomechanical design and periodontal health maintenance, thorough assessment of pre-treatment alveolar bone features is also crucial for the prevention of further bone loss during treatment. Distalization of dentition to avoid excessive labial tipping of the mandibular anterior teeth or augmentation of the bone housing should be considered, particularly in patients with high facial divergence. Lateral ridge augmentation with corticotomy of the mandibular anterior teeth increases the labial bone thickness directly, thus allowing a greater amount of labial movement, and lessening the decrease in alveolar bone height, implying the potential of reducing the risk of dehiscence/ fenestration^38,39^. In addition to accelerating tooth movement, this procedure can protect alveolar bone housing from the risk of gingival recessions when substantial proclination of the mandibular incisors is needed.

This retrospective study had some limitations. The investigation of treatment changes focused only on hard tissue, not the overlying soft tissue. The assessment of alveolar bone thickness was limited to the midsagittal plane at the level of the root apex. The periodontal health of the mandibular anterior teeth could be more comprehensively assessed by combining clinical observation of gingival recession and topographic examination of CBCT for bone fenestration and dehiscence.

## Conclusion

The alveolar bone thickness and height of the mandibular anterior teeth were smaller in the high-MPA patients than in the low-MPA patients. All of the lingual thickness, total thickness, labial height, and lingual height of the alveolar bone significantly decreased after presurgcial orthodontic treatmnet, regardless of the extent of facial divergence. The multivariate linear mixed-effect models revealed five factors significantly affecting alveolar bone dimensions during decompensation, including facial divergence, incisor irregularity, tooth site, treatment time, and change in proclination. Collectively, the results of this study provide additional evidence to help more comprehensive consideration for the surgical-orthodontic treatment of Class III patients. The pre-surgical orthodontic treatment to decompensate mandibular anterior teeth should be approached very carefully in all MPA groups.

## Supplementary information

Supplementary Figure S1.

Supplementary Figure S2.

Supplementary Legends.

## References

[1] Capelozza L, Martins A, Mazzotini R, Silva O (1996). Effects of dental decompensation on the surgical treatment of mandibular prognathism. Int. J. Adult. Orthodon. Orthognath. Surg..

[2] Johnston C, Burden D, Kennedy D, Harradine N, Stevenson M (2006). Class III surgical-orthodontic treatment: a cephalometric study. Am. J. Orthod. Dentofac. Orthop..

[3] Troy BA, Shanker S, Fields HW, Vig K, Johnston W (2009). Comparison of incisor inclination in patients with Class III malocclusion treated with orthognathic surgery or orthodontic camouflage. Am. J. Orthod. Dentofac. Orthop..

[4] Ozdemir F, Tozlu M, Germec-Cakan D (2013). Cortical bone thickness of the alveolar process measured with cone-beam computed tomography in patients with different facial types. Am. J. Orthod. Dentofac. Orthop..

[5] Ahn HW, Baek SH (2011). Skeletal anteroposterior discrepancy and vertical type effects on lower incisor preoperative decompensation and postoperative compensation in skeletal Class III patients. Angle. Orthod..

[6] Horner KA, Behrents RG, Kim KB, Buschang PH (2012). Cortical bone and ridge thickness of hyperdivergent and hypodivergent adults. Am. J. Orthod. Dentofac. Orthop..

[7] Swasty D (2011). Cross-sectional human mandibular morphology as assessed in vivo by cone-beam computed tomography in patients with different vertical facial dimensions. Am. J. Orthod. Dentofac. Orthop..

[8] Chung CJ, Jung S, Baik HS (2008). Morphological characteristics of the symphyseal region in adult skeletal Class III crossbite and openbite malocclusions. Angle Orthod..

[9] Qu X (2017). Dentofacial traits in association with lower incisor alveolar cancellous bone thickness: a multiple regression analysis. Angle Orthod..

[10] Jäger F, Mah JK, Bumann A (2017). Peridental bone changes after orthodontic tooth movement with fixed appliances: a cone-beam computed tomographic study. Angle Orthod..

[11] Yu Q, Pan XG, Ji GP, Shen G (2009). The association between lower incisal inclination and morphology of the supporting alveolar bone: a cone-beam CT study. Int. J. Oral Sci..

[12] Sendyk M, de Paiva JB, Abrao J, Rino NJ (2017). Correlation between buccolingual tooth inclination and alveolar bone thickness in subjects with Class III dentofacial deformities. Am. J. Orthod. Dentofac. Orthop..

[13] Srebrzynska-Witek A, Koszowski R, Rozylo-Kalinowska I (2018). Relationship between anterior mandibular bone thickness and the angulation of incisors and canines: a CBCT study. Clin. Oral Investig..

[14] Sun BY, Tang J, Xiao PYD (2015). (2015) Presurgical orthodontic decompensation alters alveolar bone condition around mandibular incisors in adults with skeletal Class III maloclusion. Int. J. Clin. Exp. Med..

[15] Lee S (2018). Assessment of lower incisor alveolar bone width using cone-beam computed tomography images in skeletal Class III adults of different vertical patterns. Korean J. Orthod..

[16] Kim Y, Park JU, Kook YA (2009). Alveolar bone loss around incisors in surgical skeletal Class III patients. Angle Orthod..

[17] Periago DR (2008). Linear accuracy and reliability of cone beam CT derived 3-dimensional Images constructed using an orthodontic volumetric rendering program. Angle Orthod..

[18] Sun Z (2010). Effect of bone thickness on alveolar bone-height measurements from cone-beam computed tomography images. Am. J. Orthod. Dentofac. Orthop..

[19] Menezes CC, Guilherme JG, Massaro CS (2016). Precision, reproducibility, and accuracy of bone crest level measurements of CBCT cross sections using different resolutions. Angle Orthod..

[20] Kim BC (2010). Integration accuracy of digital dental models and 3-dimensional computerized tomography images by sequential point- and surface-based markerless registration.. Oral Surg. Oral Med. Oral Pathol. Oral Radiol. Endod..

[21] Noh H, Nabha W, Cho JH, Hwang HS (2011). Registration accuracy in the integration of laser-scanned dental images into maxillofacial cone-beam computed tomography images. Am. J. Orthod. Dentofac. Orthop..

[22] Lee KM, Kim YI, Park SB, Son WS (2012). Alveolar bone loss around lower incisors during surgical orthodontic treatment in mandibular prognathism. Angle Orthod..

[23] Kook YA, Kim G, Kim Y (2012). Comparison of alveolar bone loss around incisors in normal occlusion samples and surgical skeletal class III patients. Angle Orthod..

[24] Handelman CS (1996). The anterior alveolus: its importance in limiting orthodontic treatment and its influence on the occurrence of iatrogenic sequelae. Angle Orthod..

[25] Kim YS, Cha JY, Yu HS, Hwang CJ (2010). Comparison of mandibular anterior alveolar bone thickness in different facial skeletal types. Korean J. Orthod..

[26] Molina-Berlanga N, Llopis-Perez J, Flores-Mir C, Puigdollers A (2013). Lower incisor dentoalveolar compensation and symphysis dimensions among Class I and III malocclusion patients with different facial vertical skeletal patterns. Angle Orthod..

[27] Eraydin F, Germec-Cakan D, Tozlu M, Ozdemir FI (2018). Three-dimensional evaluation of alveolar bone thickness of mandibular anterior teeth in different dentofacial types. Niger. J. Clin. Pract..

[28] Chang ZC (2013). How to achieve proper overbite: lessons from natural dentoalveolar compensation. J. Dent. Sci..

[29] Chen YJ (2016). A new classification of mandibular asymmetry and evaluation of surgical-orthodontic treatment outcomes in Class III malocclusion. J. Craniomaxillofac. Sur..

[30] Bazina M (2018). Precision and reliability of dolphin 3-dimensional voxel-based superimposition. Am. J. Orthod. Dentofac. Orthop..

[31] Kim YI, Choi YK, Park SB, Son WS, Kim SS (2012). Three-dimensional analysis of dental decompensation for skeletal Class III malocclusion on the basis of vertical skeletal patterns obtained using cone-beam computed tomography. Korean J. Orthod..

[32] Little RM (1975). The irregularity index: a quantitative score of mandibular anterior alignment. Am. J. Orthod..

[33] Yamada C (2007). Spatial relationships between the mandibular central incisor and associated alveolar bone in adults with mandibular prognathism. Angle Orthod..

[34] Park JH, Hong JY, Ahn HW, Kim SJ (2018). Correlation between periodontal soft tissue and hard tissue surrounding incisors in skeletal Class III patients. Angle Orthod..

[35] Patcas R, Müller L, Ullrich O, Peltomäki T (2012). Accuracy of cone-beam computed tomography at different resolutions assessed on the bony covering of the mandibular anterior teeth. Am. J. Orthod. Dentofac. Orthop..

[36] Garib DG, Yatabe MS, Ozawa TO, Filho OGdS (2010). Alveolar bone morphology under the perspective of the computed tomography: defining the biological limits of tooth movement. Dental Press J. Orthod..

[37] Nimigean V (2009). (2009) Alveolar bone dehiscences and fenestrations: an anatomical study and review. Rom. J. Morphol. Embryol..

[38] Ahn HW (2016). Morphologic evaluation of dentoalveolar structures of mandibular anterior teeth during augmented corticotomy-assisted decompensation. Am. J. Orthod. Dentofac. Orthop..

[39] Coscia G, Coscia V, Peluso V, Addabbo F (2013). Augmented corticotomy combined with accelerated orthodontic forces in class III orthognathic patients: morphologic aspects of the mandibular anterior ridge with cone-beam computed tomography. J. Oral. Maxillofac. Surg..

