# Supplementary figures and images for: Architectural changes in alveolar bone for dental decompensation before surgery in Class III patients with differing facial divergence: a CBCT study
Source: Sci Rep. 2020 Sep 1;10:14379. doi: 10.1038/s41598-020-71126-3 (PMC7463229; doi:10.1038/s41598-020-71126-3)

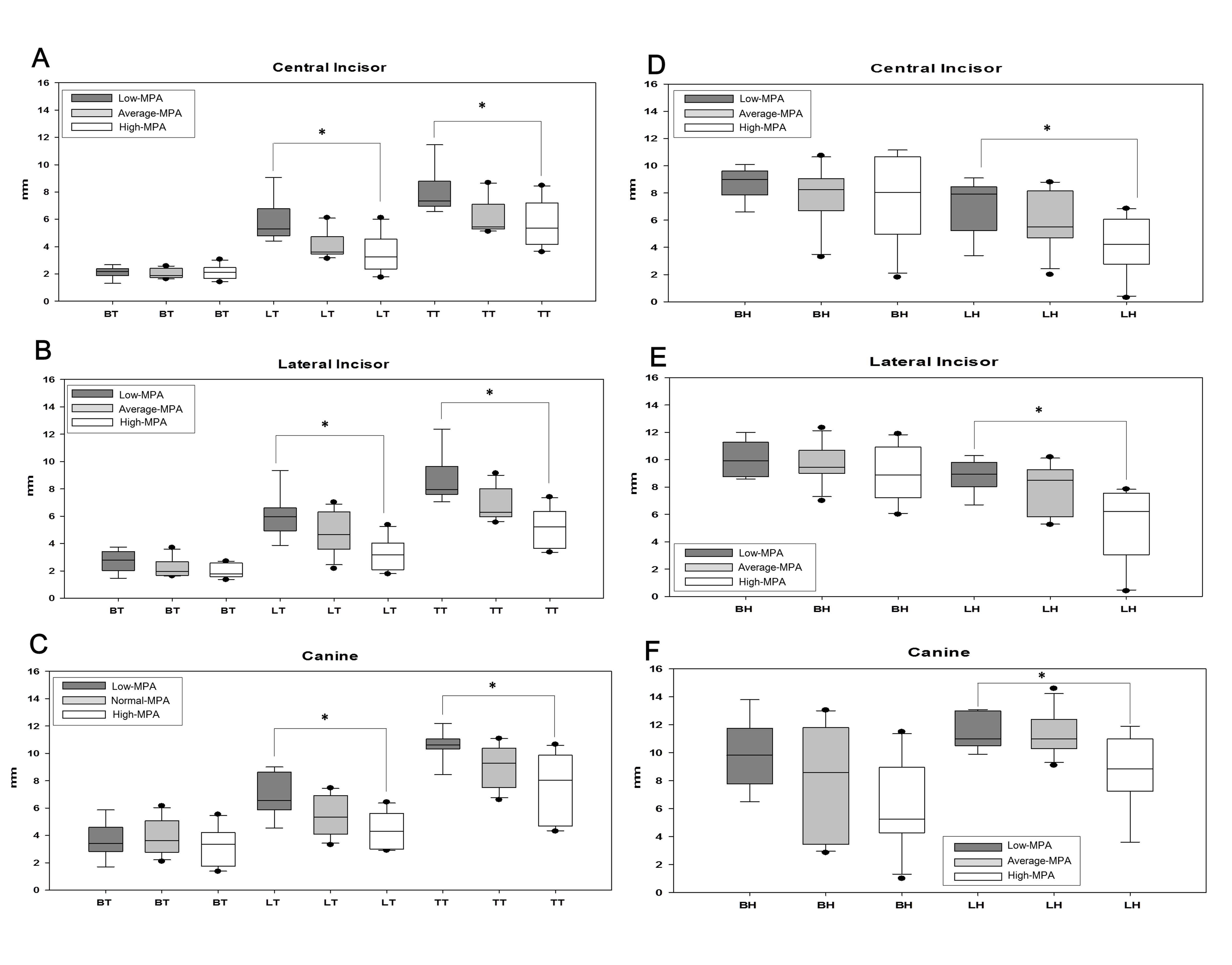

Supplement: Supplementary file 1 — Supplementary Figure S1. [file 41598_2020_71126_MOESM1_ESM.tif]

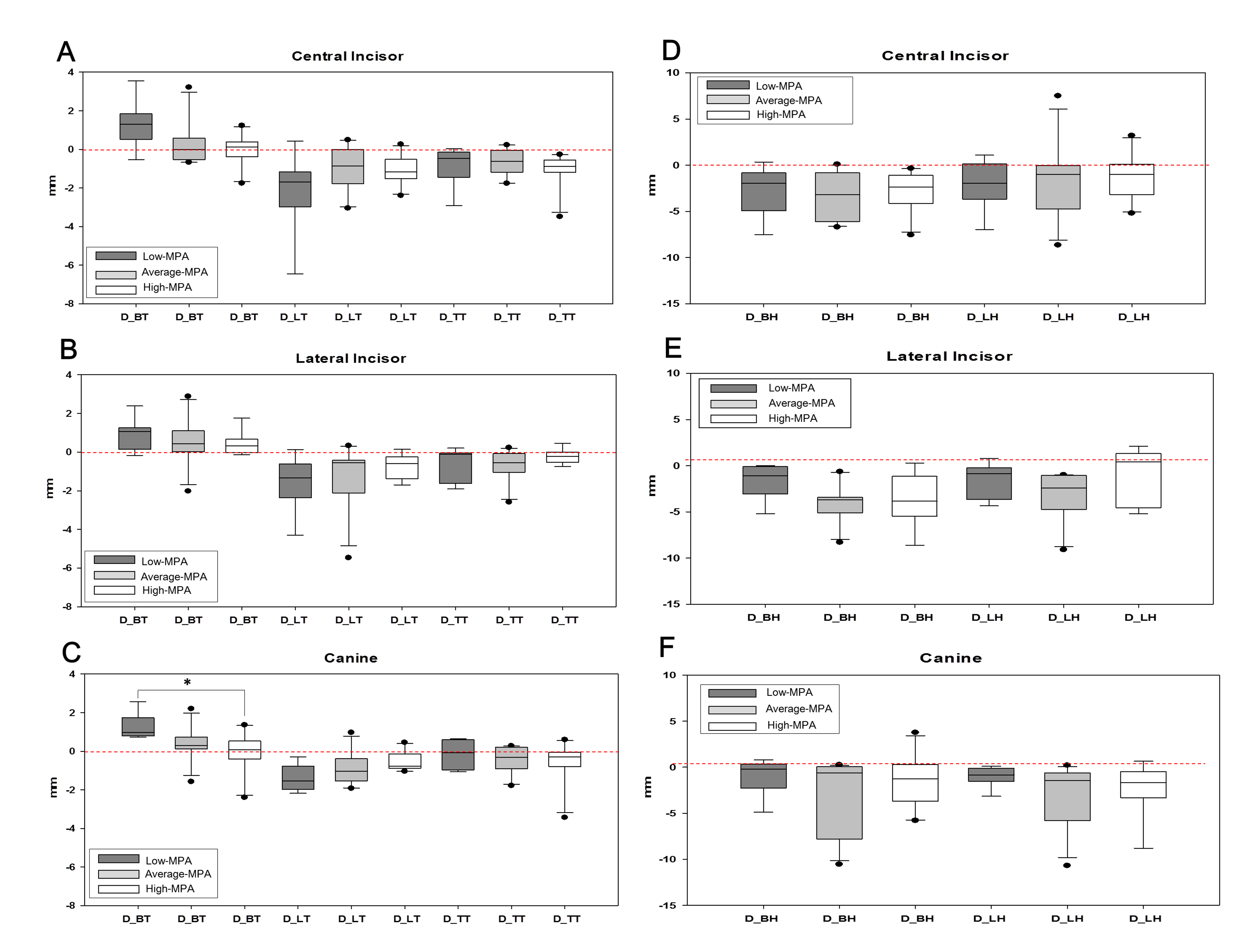

Supplement: Supplementary file 2 — Supplementary Figure S2. [file 41598_2020_71126_MOESM2_ESM.tif]
