# Supplementary material for: Architectural changes in alveolar bone for dental decompensation before surgery in Class III patients with differing facial divergence: a CBCT study
Source: Sci Rep. 2020 Sep 1;10:14379. doi: 10.1038/s41598-020-71126-3 (PMC7463229; doi:10.1038/s41598-020-71126-3)
Supplement: Supplementary file 3 — Supplementary Legends. [file 41598_2020_71126_MOESM3_ESM.docx]

**Legends for supplementary figures**

Supplementary Figure 1

Boxplots showing the alveolar bone dimensions of mandibular anterior teeth in the three facial divergence groups.

A, B, C: The alveolar bone thickness of the central incisors, lateral incisors, and canines.

D, E, F: The alveolar bone height of the central incisors, lateral incisors, and canines.

**^*^** Significant difference between groups (based on the Kruskal-Wallis test, *p*<0.05).

Supplementary Figure 2

Boxplots showing the differences in alveolar bone dimensions after presurgical decompensation in the three facial divergence groups.

A, B, C: Differences in the alveolar bone thickness of the central incisors, lateral incisors, and canines.

D, E, F: Differences in the alveolar bone height of the central incisors, lateral incisors, and canines. Greater variation was noted in the measurement of alveolar height (D_BH, D_LH) compared to alveolar thickness (D_BT, D_LT, and D_TT).

The Kruskal-Wallis test showed a significant difference in the measurement D_BT of the canines between the low-MPA and high-MPA groups.

**^*^** *p*<0.05.
